# Dietary Intake of Polyphenols and All-Cause Mortality: A Systematic Review with Meta-Analysis

**DOI:** 10.3390/metabo14080404

**Published:** 2024-07-25

**Authors:** Roberta Zupo, Fabio Castellana, Giuseppe Lisco, Filomena Corbo, Pasquale Crupi, Rodolfo Sardone, Francesco Panza, Madia Lozupone, Mariangela Rondanelli, Maria Lisa Clodoveo

**Affiliations:** 1 Department of Interdisciplinary Medicine (DIM), University of Bari Aldo Moro, Piazza Giulio Cesare 11, 70100 Bari, Italy; roberta.zupo@uniba.it (R.Z.); fabio.castellana@uniba.it (F.C.); giuseppe.lisco@uniba.it (G.L.); 2 Department of Pharmacy-Drug Sciences, University of Bari “Aldo Moro”, 70125 Bari, Italy; filomena.corbo@uniba.it; 3 Department of Agricultural, Food and Forest Science, University of Palermo, Viale delle Scienze, 90128 Palermo, Italy; pasquale.crupi@unipa.it; 4 Unit of Statistics and Epidemiology, Local Health Authority of Taranto, 74121 Taranto, Italy; rodolfo.sardone@asl.taranto.it; 5 “Cesare Frugoni” Internal and Geriatric Medicine and Memory Unit, Department of Interdisciplinary Medicine (DIM), University of Bari Aldo Moro, Piazza Giulio Cesare 11, 70100 Bari, Italy; f_panza@hotmail.com; 6 Department of Translational Biomedicine and Neuroscience “DiBraiN”, University of Bari Aldo Moro, 70121 Bari, Italy; madia.lozupone@gmail.com; 7 Department of Public Health, Experimental and Forensic Medicine, University of Pavia, 27100 Pavia, Italy; mariangela.rondanelli@unipv.it

**Keywords:** polyphenols, antioxidants, diet, mortality, meta-analysis, systematic review

## Abstract

Polyphenols are secondary metabolites found in plants, foods, and drinks, occurring in small quantities and showcasing antioxidant and anti-inflammatory qualities. The primary polyphenols consist of flavonoids, phenolic acids, stilbenes, and lignans. However, there is currently no comprehensive quantitative analysis of epidemiological data on overall death rates. This systematic review with meta-analysis aims to identify the exposure–response relationship between dietary polyphenol intake and all-cause mortality. The literature was reviewed from its earliest study to May 2024, utilizing six distinct electronic databases. No specific criteria were used to choose participants based on the recruiting environment, their general health condition, country, or ethnicity. The inclusion criteria for studies were as follows: a longitudinal design, exposure to dietary polyphenols, all-cause mortality as the outcome, and hazard risk (HR) as the impact measure. The Newcastle–Ottawa Scale was used to evaluate the methodological rigor of the study. The hazard risks (HRs) and 95% confidence intervals (CIs) were estimated by pooling data using common effects models. A protocol has been registered on PROSPERO with the identification number CRD42024545524. The meta-analysis comprised seven cohort studies that involved 178,657 adult people aged 18 years and older. These studies examined the relationship between total dietary polyphenol consumption and the risk of all-cause death. The recruitment settings exclusively used community-based approaches, with a preference for Europe (71%) in terms of geographic distribution. The study’s quality was assessed to be moderate to high. The meta-analysis showed consistent evidence that increased dietary exposure to polyphenols reduces the risk of all-cause mortality by 7% (HR 0.93, 95% CI 0.91–0.95, *I*^2^: 48%). Pooled data from the available evidence consistently show that individuals exposed to an antioxidant diet rich in polyphenol sources may be at lower risk of all-cause mortality.

## 1. Introduction

Nutrition and lifestyle are key modifiable factors in preventing chronic non-communicable diseases and substantially reducing all-cause mortality in most Westernized countries. The Mediterranean diet (MedDiet), a well-characterized dietary pattern, has been indicated to be associated with longevity and better quality of life, reducing the risk of the most common chronic diseases such as cardiovascular disease (CVD), metabolic syndrome, age-related cognitive impairment, physical frailty, type 2 diabetes mellitus (T2DM), osteoporosis, cancer, and also all-cause mortality [1,2,3,4,5,6,7]. By definition, the MedDiet eating pattern abounds in fruits and vegetables, olive oil, nuts, legumes, whole grain bread, and fish, while wine is consumed in moderate amounts during meals [7]. 

Bromatologically, the MedDiet is greatly rich in mono- and polyunsaturated fatty acids [8] and also polyphenols, which are bioactive compounds found mainly in plant foods and plant-derived beverages, such as coffee, tea, and red wine [9,10]. Some polyphenols contribute to the specific color [11] and sensory characteristics of foods [12]. Polyphenols are also one of the most abundant dietary bioactive compounds [13] in plants, and unlike vitamins and minerals, low intake of them is not linked to deficiency diseases, but adequate consumption has beneficial effects on health [14]. Based on their chemical structure, polyphenols are classified into four main groups: flavonoids, phenolic acids, stilbenes, and lignans [15].

A large body of research has found polyphenols to be beneficial to health due to their antioxidant [16] and anti-inflammatory [17] effects, which also include antihypertensive [18] and antidiabetic [19] properties. As a result, polyphenols have been associated with a lower risk of chronic diseases, such as CVD [14,20,21], diabetes [14,22], and some cancers, including colorectal [23] and breast cancer [23].

As polyphenol intake may protect against the development of chronic diseases, such as CVD, cancer, and T2DM, an increased consumption of polyphenols is speculated to play a role in lowering the risk of all-cause mortality while providing a longer life expectancy.

To date, some studies have described the association between specific groups of polyphenols and mortality [24,25,26], while others have examined the total intake of polyphenols in association with all-cause mortality. However, as yet, no pooled data have estimated an exposure–response function between dietary polyphenol consumption and all-cause mortality. This research aims to provide a systematic review with a meta-analysis of observational cohort studies investigating the association between total dietary polyphenol consumption and all-cause mortality.

## 2. Methods

### 2.1. Search Strategy and Data Extraction

The present systematic review followed the Preferred Reporting Items for Systematic Reviews and Meta-Analyses (PRISMA) guidelines, adhering to the PRISMA 27-item checklist [27]. An a priori protocol for the search strategy and inclusion criteria was established and registered, without particular amendments to the information provided at registration, on PROSPERO as a prospective international register of systematic reviews (CRD42024545524). We performed separate searches in the US National Library of Medicine (PubMed), Medical Literature Analysis and Retrieval System Online (MEDLINE), EMBASE, Scopus, Ovid, and Google Scholar databases to find original articles inquiring into any association between the exposure to total daily dietary polyphenols and all-cause mortality as the outcome. Thus, the main objective was to evaluate the association between exposure to a cluster of foods polyphenols, as assessed by dietary intake or urinary concentration, and all-cause mortality in the general population. We also considered grey literature using the largest archive of preprints https://arxiv.org/ in the study selection phase and http://www.opengrey.eu/ database to access remarkable conference abstracts and other non-peer-reviewed material. Since we chose only observational studies to be included, the search strategy followed PECO (populations, exposure, comparator, and outcome) concepts [28], including populations (humans), exposure (total dietary polyphenols), comparators (exposure levels), and outcome (all-cause mortality). 

The search strategy used in PubMed and MEDLINE and adapted to the other four electronic sources is detailed in Table 1. In the literature search, no time limit was set, and articles were retrieved until 31 May 2024. No language limitation was introduced. Two researchers (RZ, FC) conducted a thorough search of publications, individually assessed the titles and abstracts of retrieved articles, cross-checked complete texts, and made selections for inclusion in the study. Technical reports, letters to the editor, and systematic and narrative review papers were not included. Inter-rater reliability (IRR) was employed to gauge the level of agreement amongst coders, and subsequently, the κ statistic was utilized as a metric to assess accuracy and precision. According to PRISMA ideas and quality evaluation procedures, a coefficient k of at least 0.9 was achieved in all data extraction stages [29].

### 2.2. Inclusion Criteria, Data Extraction, and Registration

The exposure and outcome were reported to humans without age restriction. No criterion was applied to the recruitment settings or health status of the study population (general population or groups with specific characteristics). Potentially eligible articles were identified by reading the abstract and, if eligible, reading the full-text version of the articles. For each article selected, the best statistical approach to confounding was considered in evaluating the magnitude of the effect size for associations. Data were cross-checked, any discrepancies were discussed, and disagreements were resolved by a third investigator (RS).

The following information was extracted by two investigators (RZ, FC) separately and in duplicate in a piloted form: (1) general information about single studies (author, year of publication, country, settings, recruitment period, design, and sample size); (2) exposure; (3) outcome; (4) covariates adjusted for; (5) follow-up period; and (6) effect of the measure on the association between exposure and outcome.

All references selected for retrieval from the databases were managed with the MS Excel software platform (version 18.0, 2021) for data collection by a biostatistician (FC). Lastly, data extracted from selected studies and stored in the database were structured as evidence tables.

### 2.3. Risk of Bias

The quality of the included studies was appraised using the Newcastle–Ottawa Quality Assessment Scale (NOS) for cohort studies [30]. Two researchers (RZ, FC) independently extracted the data and conducted the study quality assessment, with consensus reached through discussion or third-party arbitration (MLC). The reviewers themselves independently analyzed the references of all evaluated articles to avoid the possible exclusion of additional studies. Any disagreement between the two researchers was resolved by discussion until a consensus was reached. The NOS evaluates the methodological quality of the studies in eight items for cohort studies within three categories, i.e., (1) the selection of participants (maximum 4 scores); (2) comparability of subjects (maximum 2 scores); and (3) assessment of outcome (maximum 3 scores). The quality of each study is classified as follows: (i) good quality if a study obtains 3 or 4 points in the selection part AND 1 or 2 points in the comparability part AND 2 or 3 points in the outcome part; (ii) fair quality if a study obtains 2 scores in the selection part AND 1 or 2 scores in the comparability part AND 2 or 3 points in the outcome part; and (iii) poor quality if a study scores 0 or 1 in the selection part OR 0 stars in the comparability part OR 0 or 1 star in the outcome part [30]. Because of the longitudinal design of the studies considered, we did not consider the “Comparability” section and question 2 of the “Selection” section (“selection of the unexposed cohort”); as such, the maximum score awarded could have been 6 instead of 9.

### 2.4. Meta-Analysis

A meta-analysis for Cox’s regression models was performed using the fully adjusted model for each included study. We considered HRs and their 95% CIs as effect sizes for the present study. Summary HRs of all-cause mortality were calculated for the highest versus lowest category of dietary polyphenol consumption using the common effects model. The derSimonian–Laird estimator was adopted as the tau method calculation. Heterogeneity was assessed by Cochran’s Q statistic and quantified (*I*^2^) [31]. The *I*^2^ statistic and *p* value were used to analyze heterogeneity among the studies. *I*^2^ values ≤ 25%, ≤50%, ≤75%, and >75% indicated no, little, moderate, and significant heterogeneity, respectively. Fixed effects models were chosen according to low heterogeneity among the included studies. A sensitivity analysis, in which one study at a time was excluded, was performed to assess the stability of results and potential sources of heterogeneity. To this aim, a leave-one-out (LOOM) meta-analysis was undertaken, which is the elimination of one research study at a time to assess the robustness of the primary results and the influence of each relationship on effect or heterogeneity. A common effects model was applied for all analyses. 

Publication bias was unable to be assessed since the meta-analysis included fewer than 10 studies for comparison [32]. Therefore, no downgrades for publication bias were made.

The analyses were conducted using Review Manager (RevMan) version 5.2 software developed by The Nordic Cochrane Centre, The Cochrane Collaboration, and R software version 223.03.1 by a senior biostatistician (FC). Significance was attributed to *p*-values below 0.05.

## 3. Results

The first systematic literature search generated 648 entries. After eliminating duplicates, 234 documents were identified as potentially relevant and chosen for their abstract and title analysis. Then, 227 studies were excluded because they did not meet the characteristics of the approach or the objective of the review. After reviewing the full text of the remaining papers, only seven met the inclusion criteria and were included in the final qualitative and quantitative analysis [33,34,35,36,37,38,39].

**Figure 1:**
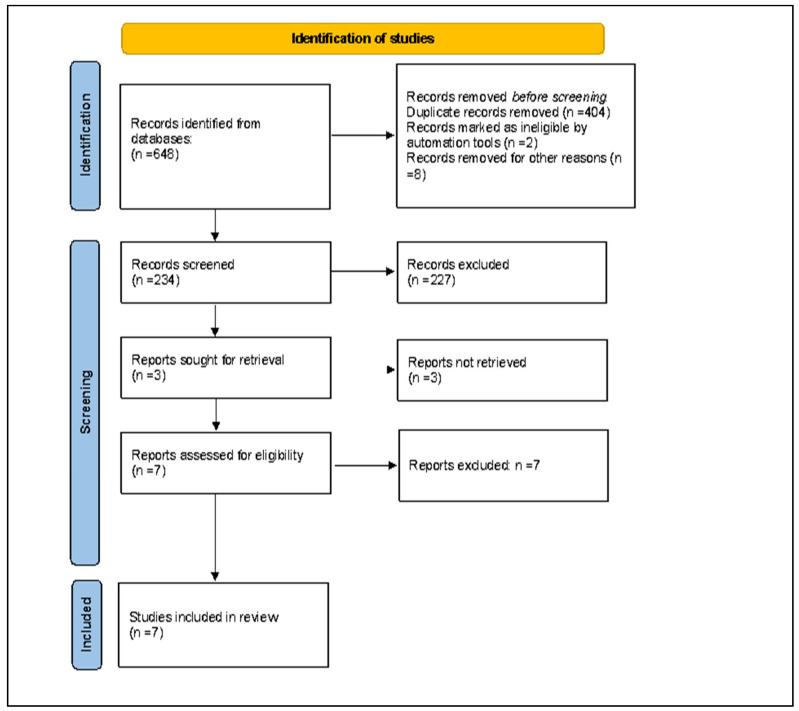
Preferred Reporting Items for Systematic Reviews and Meta-Analyses (PRISMA) flow chart illustrating the number of studies at each stage of the review.

The flow chart of the Preferred Reporting Items for Systematic Reviews and Meta-Analyses (PRISMA), illustrating the number of studies in each stage of the review, is shown in Figure 1. The final study base included seven original articles reporting observational studies examining the association between total dietary polyphenol exposure and all-cause mortality. 

Table 2 shows descriptive data extracted and related to the study design, sample size (N), recruitment period, sex (if reported), study population, country, follow-up period, effect measure, and covariates adjusted for. All studies featured an observational cohort design, with an average follow-up of 4 years (range 5–12 years). The recruitment settings were all community-based, and the geographic distribution of studies found Europe (71%, N = 5) to be predominant, with a minority from Asia (14.5%, N = 1) and America (14.5%, N = 1).

The total population included 178657 adult individuals (18+ years). The exposure assessment was carried out by calculating daily dietary polyphenols from the food frequency questionnaires (FFQs) being validated on the study populations and employing tools such as the Phenol-Explorer dataset, i.e., the first comprehensive database on polyphenol content in foods or the U.S. Department of Agriculture dataset (USDA) [40], or the phenolic antioxidant coefficient (PAC) score, i.e., a scoring system that assesses polyphenol intake with a seven-item score by generating intake deciles for the class and subclass of polyphenols included (flavonols, anthocyanins, flavanones, flavones, flavanols, isoflavonoids, and lignans). The only exception was the report by Zamora-Ros and colleagues, who used urinary polyphenol concentration as a proxy [26]. Regarding the effect measure estimates, all studies consistently reported the hazard ratio (HR) with 95% CIs for all-cause mortality according to the tertiles [37] or quartiles [33,34,35] or quintiles [37,38] of polyphenol consumption.

**Figure 2:**
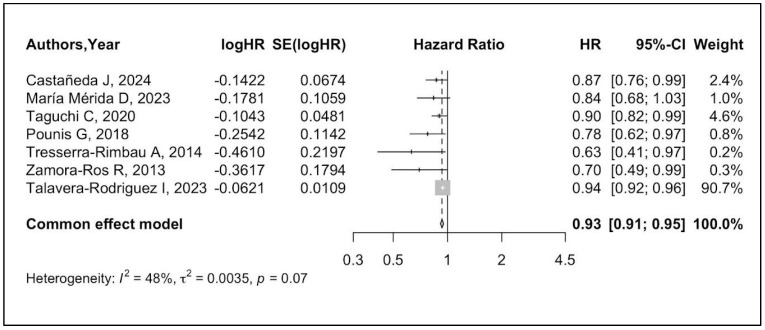
Forest plot for the association between dietary polyphenols and all-cause mortality, grouping data from 7 studies [33,34,35,36,37,38,39]. The diamond indicates the pooled HR estimates with 95% CI. Abbreviations: CI, confidence interval; HR, hazard ratio.

**Figure 3:**
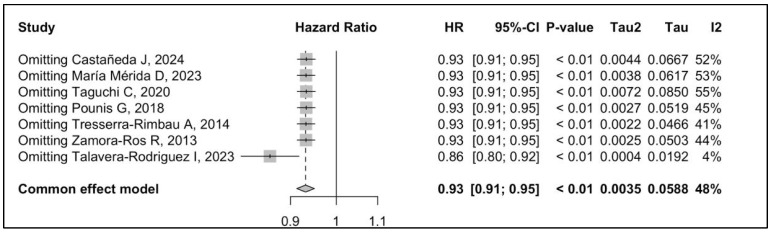
Sensitive analysis of dietary polyphenol consumption (highest versus lowest category) and all-cause mortality [33,34,35,36,37,38,39].

The pooled effect size of the association between dietary polyphenol intake and all-cause mortality is summarized in Figure 2. The meta-analysis shows strong and consistent evidence that higher dietary exposure to polyphenols reduces the risk of all-cause mortality by 7% (HR 0.93, 95% CI 0.91–0.95, *I*^2^: 48%) after maximum adjustment for confounders. The quality assessment of the seven studies according to the NOS showed an average high level of quality, with the majority of studies scoring between 5 and 6 (Table 3). Regarding the robustness of the overall effect sizes, we conducted LOOM analysis for sensitivity. The results of the sensitivity analysis showed that, after excluding each study at a time, higher daily dietary polyphenol consumption was still largely associated with a significant reduction in all-cause mortality risk, indicating that the results of our meta-analysis are robust (Figure 3).

## 4. Discussion

The present systematic review addresses the conceptual hypothesis of a link between dietary antioxidant load, as assessed using total dietary polyphenol intake, and the hazard of all-cause mortality in the adult population. Therefore, a systematic review with a meta-analysis of existing observational cohort studies investigating the association between total dietary polyphenol consumption and all-cause mortality was provided, concluding that consistency exists in the direction of an association and that higher dietary exposure to polyphenols leads to a 7% reduced risk of all-cause mortality (HR 0.93, 95% CI 0.91–0.95, *I*^2^: 48%).

All epidemiological evidence on the cluster of etiopathogenesis factors associated with chronic diseases has so far supported the hypothesis that an increased intake of polyphenols and the many subclasses of polyphenols they represent have the potential to extend longevity through multifactorial pathways. 

Several studies are being discussed, which hereafter may corroborate possible biological pathways. Of these mechanisms, one includes the anti-atherosclerotic potential of some polyphenols and their metabolites, which can act beneficially in enhancing endothelial function and antioxidant status, boosting nitric oxide release and modulating inflammation and lipid metabolism to reduce cardiovascular health outcomes [41,42,43,44,45]. There is also evidence that polyphenols may act as chemo-preventive agents. In this regard, resveratrol is a well-known stilbene, found mainly in red wine and grapes, described for its many health benefits, including the inhibition of tumorigenesis [46,47,48,49]. In vitro and in vivo studies have shown that epigallocatechin-3-gallate, the main polyphenol in green tea, exerts anticarcinogenic effects, such as the inhibition of growth proliferation, the induction of apoptosis and phase II detoxifying enzymes, and a reduction in oxidative DNA damage [49]. Xanthohumol, quercetin, curcumin, and genistein are other examples of polyphenols that have demonstrated anticarcinogenic properties through their ability to inhibit tumor growth [47,49,50,51]. Other prominent phenolic compounds include hydroxytyrosol, oleuropein, oleocanthal, and lignans, known as the main bioactives of extra virgin olive oil, which have proven anti-inflammatory, metabolic and chemo-preventive effects, especially in the contexts of the Mediterranean diet [9,10,52,53]. 

Moreover, available evidence on dietary polyphenols also supports their role in reducing the risk of type 2 diabetes mellitus (T2DM), another high-incidence chronic non-communicable disease. In this regard, data from three U.S. prospective cohorts concluded that anthocyanins are inversely associated with the risk of T2DM, and similar associations were found for blueberries, grapes, and apples [39,40]. Finally, polyphenols have been proposed as promising phytochemicals for the treatment and prevention of neurogenerative diseases such as Alzheimer’s disease, Parkinson’s disease, and other neurological disorders that should not be underestimated from the perspective of the aging demographic [29,41].

Although little is known about the trajectories of the interactive or synergistic actions of polyphenols, it is necessary to speculate that a combination of diet and lifestyle-related antioxidant systems may have greater beneficial effects than a single factor alone or even a sum of isolated factors. That is, the total load of polyphenols could exert a greater effect than the sum of its parts. Thus, since several factors may influence multiple pathways, lifestyle and diet may act synergistically in prolonging life expectancy, possibly reducing intermediate risk factors such as high-incidence chronic diseases that may shorten life expectancy [54]. Moreover, the relationship between oxidative stress and inflammation is well-established, and both processes are linked to the development of non-communicable diseases and reduced survival, hence why polyphenols act sufficiently in counteracting mortality trajectories.

## 5. Strengths and Limitations

The main strength of our study is the inclusion of prospective cohort studies and a large number of participants and deaths, providing greater statistical power to quantitatively assess the association between dietary polyphenol consumption and all-cause mortality. We also conducted meta-analyses of the pooled data to clarify the strength and shape of the observed associations. Other strengths include the use of a comprehensive search strategy, sensitivity and effects, the assessment of risk of bias, and certainty of evidence for each association.

Our findings should be interpreted considering several limitations. First, because of the observational nature of the included studies, the reported associations may be influenced by residual or unmeasured confounding factors, although many covariates were considered. Further, the use of FFQ to assess daily dietary intake, although a common and validated method in epidemiological research, suffers from a reporting bias related to self-perceived intakes. To sum up, the present meta-analysis contributes to the literature on dietary polyphenols and mortality, which has so far lacked a qualitative and quantitative synthesis; however, the findings should be used with caution considering the inherent limitations mentioned above.

## 6. Conclusions

Quantitative findings of pooled available data consistently provide evidence that individuals exposed to an antioxidant diet rich in polyphenol sources may have a lower risk of all-cause mortality, probably through a multifactorial biological pathway.

## Figures and Tables

**Table 1:** Search strategy used in the US National Library of Medicine (PubMed) and Medical Literature Analysis and Retrieval System Online (MEDLINE) and adapted to the other sources, according to selected descriptors.

	*Strategy*	*Descriptors Used*
#1	Population	(human*[tiab])
#2	Intervention/Exposure	(intake[tiab]) OR (consumption[tiab]) OR (exposure*[tiab])
#3	Comparator	(Categor*[tiab]) OR (exposure[tiab]) OR (tertile*[tiab]) OR (quartile*[tiab]) OR (quintile*[tiab]) OR (level*[tiab])
#4	Outcomes	(overall mortality[tiab]) OR (survival[tiab]) OR (death*[tiab]) OR (all-cause mortality[tiab])
#5	*Exclusion keywords*	(Review[tiab]) OR (systematic review[tiab]) OR (narrative review[tiab]) OR (meta-analysis[tiab]) OR (editorial[tiab]) OR (letter[tiab]) OR (commentary[tiab]) OR (perspective[tiab]) OR (book[tiab])
#6	*Search strategy*	#1 AND #2 AND #3 AND #4 NOT #5
	Filters: Sort by: most recent. Date: 31 May 2024. Time restriction: none.	

**Table 2:** Descriptive of selected studies N = 7.

Author, Year	Recruitment Period	Sex	Country	N	Study Population	Follow-Up	Design	Exposure	Outcome(s)	Covariates Adjusted for
Castañeda J et al., 2024 [33]	2008–2011	All female	America (Mexico)	95,313	The Mexican Teachers’ Cohort Study	11.2 years	cohort	Dietary polyphenols	All-cause mortality	Education, smoking status, leisure time and household physical activity, time watching TV, former drinker status, Body Mass Index (BMI), energy intake, fiber intake, number of medications per day, number of chronic diseases, age, sex, hypertriglyceridemia, hypercholesterolemia, low HDL-cholesterol, and hypertension.
Talavera-Rodriguez I et al., 2023 [35]	1999–2009	7580 (M), 10,981 (F), aged ≥20 years	Europe (Spain)	18,561	The Seguimiento Universidad de Navarra (SUN) Study	12.2 years	cohort	Dietary polyphenols	All-cause mortality	Age, family history of cardiocascular disease (CVD), following special diet at baseline, marital status, Mediterranean adherence, prevalent cancer, depression, CVD, diabetes, dyslipidemia, hypertension, sex, total energy intake, use of aspirin, education.
María Mérida D et al., 2023 [34]	2008–2010	5760 (M), 6401 (F), aged ≥18 years	Europe (Spain)	12,161	The Study on Nutrition and Cardiovascular Risk (ENRICA)	12.5 years	cohort	Dietary polyphenols	All-cause mortality	Age, sex, education, smoking status, leisure time and household physical activity, time watching TV, former drinker, BMI, total energy intake, total fiber intake, number of medications per day, number of chronic conditions, hypertriglyceridemia, hypercholesterolemia, low HDL-cholesterol, and hypertension.
Taguchi C et al., 2020 [36]	1992–2008	14,427 (M), 17,125 (F), aged ≥35 years	Asia (Japan)	29,079	The Takayama study	16 years	cohort	Dietary polyphenols	All-cause mortality	Age, sex, total energy, BMI, physical activity, smoking status, education, marital status, histories of diabetes and hypertension, alcohol consumption, and intake of salt.
Pounis G et al., 2018 [37]	2005–2010	10,980 (F), 10,322 (M), aged ≥35 y	Europe (Italy)	21,302	The Moli-sani study	8.3 years	cohort	Dietary polyphenols	All-cause mortality	Age, energy intake, smoking habits, social status, physical activity level, and the low-grade inflammation status of the participants assessed through the INFLA score.
Tresserra-Rimbau A et al., 2014 [38]	2003–2010	NR	Europe (Spain)	1434	The PREDIMED trial	4.8 years	cohort	Dietary polyphenols	All-cause mortality	Age, smoking, BMI, baseline diabetes, alcohol, total energy intake, physical activity, family history of CVD or cancer, aspirin use, antihypertensive drug use, use of cardiovascular medication, use of oral hypoglycaemic agents, insulin, intake of protein, saturated fatty acids, polyunsaturated fatty acids, monounsaturated fatty acids, and cholesterol.
Zamora-Ros R et al., 2013 [39]	1998–2000	444 (F), 363 (M), aged ≥65 years	Europe (Italy)	807	The INCHIANTI study	12 years	cohort	Total urinary polyphenols	All-cause mortality	Education, BMI, total energy intake, alcohol intake, smoking history, physical activity, CVD, cancer, diabetes mellitus, dementia, Parkinson’s disease, and chronic obstructive pulmonary disease.

**Table 3:** Newcastle–Ottawa Scale (NOS) for quality assessment.

Author, Year	Selection 1 (Representativeness of Exposure)	Selection 3 (Exposure Assessment)	Selection 4 (Outcome of Interest)	Outcome 1 (Outcome Ascertainment)	Outcome 2 (Duration of Follow-Up)	Outcome 3 (Adequate Follow-Up Time)	NOS Score (Maximum 6)
Castañeda J et al., 2024 [33]	*	*	*	*	*	*	6
Talavera-Rodriguez I et al., 2023 [35]	*	-	*	*	*	*	5
María Mérida D et al., 2023 [34]	*	*	*	*	*	*	6
Taguchi C et al., 2020 [36]	*	-	*	*	*	*	5
Pounis G et al., 2018 [37]	*	-	*	*	*	*	5
Tresserra-Rimbau A et al., 2014 [38]	*	-	*	*	*	-	4
Zamora-Ros R et al., 2013 [39]	-	-	*	*	*	*	5

*: value corresponding to 1 point for the score. Because of the design of the studies, we decided to use the NOS scale for cohort studies but did not consider the section for “Comparability” and question 2 in the section “Selection” (“selection of the non-exposed cohort”). For the full version of the NOS scale, see the following: http://www.ohri.ca/programs/clinical_epidemiology/oxford.asp (accessed on 15 May 2024).

## Data Availability

All data supporting the findings of this study are available from the corresponding author (MLC) upon reasonable request.
