# Correction: Zupo et al. Dietary Intake of Polyphenols and All-Cause Mortality: A Systematic Review with Meta-Analysis. *Metabolites* 2024, *14*, 404

**DOI:** 10.3390/metabo16080522

**Published:** 2026-07-24

**Authors:** Roberta Zupo, Fabio Castellana, Giuseppe Lisco, Filomena Corbo, Pasquale Crupi, Rodolfo Sardone, Francesco Panza, Madia Lozupone, Mariangela Rondanelli, Maria Lisa Clodoveo

**Affiliations:** 1 Department of Interdisciplinary Medicine (DIM), University of Bari Aldo Moro, Piazza Giulio Cesare 11, 70100 Bari, Italygiuseppe.lisco@uniba.it (G.L.); 2 Department of Pharmacy-Drug Sciences, University of Bari “Aldo Moro”, 70125 Bari, Italy; 3 Department of Agricultural, Food and Forest Science, University of Palermo, Viale delle Scienze, 90128 Palermo, Italy; 4 Unit of Statistics and Epidemiology, Local Health Authority of Taranto, 74121 Taranto, Italy; 5 “Cesare Frugoni” Internal and Geriatric Medicine and Memory Unit, Department of Interdisciplinary Medicine (DIM), University of Bari Aldo Moro, Piazza Giulio Cesare 11, 70100 Bari, Italy; f_panza@hotmail.com; 6 Department of Translational Biomedicine and Neuroscience “DiBraiN”, University of Bari Aldo Moro, 70121 Bari, Italy; madia.lozupone@gmail.com; 7 Department of Public Health, Experimental and Forensic Medicine, University of Pavia, 27100 Pavia, Italy

## Text Correction

There was an error in the original publication [1]. A correction has been made to reference [7], cited in the Introduction. Reference [7] has been replaced with the following:

Guasch-Ferré, M.; Willett, W.C. The Mediterranean Diet and Health: A Comprehensive Overview. *J. Intern. Med.* **2021**, *290*, 549–566.

The authors state that the scientific conclusions are unaffected. This correction was approved by the Academic Editor. The original publication has also been updated.
